# Metabolomic profiling of polymyxin-B in combination with meropenem and sulbactam against multi-drug resistant *Acinetobacter baumannii*

**DOI:** 10.3389/fmicb.2022.1013934

**Published:** 2022-09-23

**Authors:** Shixing Zhu, Jiayuan Zhang, Chu Song, Yuwei Liu, Charles Oo, M. Tobias Heinrichs, Zhihua Lv, Yuanqi Zhu, Sherwin K. B. Sy, Pan Deng, Mingming Yu

**Affiliations:** ^1^School of Medicine and Pharmacy, Ocean University of China, Qingdao, China; ^2^SunLife Biopharma, Morris, NJ, United States; ^3^Department of Pharmaceutics, College of Pharmacy, University of Florida, Gainesville, FL, United States; ^4^Laboratory for Marine Drugs and Bioproducts of Qingdao National Laboratory for Marine Science and Technology, Qingdao, China; ^5^Department of Laboratory Medicine, the Affiliated Hospital of Qingdao University, Qingdao, China; ^6^Department of Statistics, State University of Maringá, Paraná, Brazil; ^7^Department of Pharmaceutical Analysis, College of Pharmaceutical Sciences, Soochow University, Suzhou, China

**Keywords:** *Acinetobacter baumannii*, metabolomics, polymyxin-B, meropenem, synergistic combinations

## Abstract

Empirical therapies using polymyxins combined with other antibiotics are recommended in the treatment of *Acinetobacter baumannii* infections. In the present study, the synergistic activities of polymyxin-B, meropenem, and sulbactam as combination therapy were investigated using metabolomic analysis. The metabolome of *A. baumannii* was investigated after treatment with polymyxin-B alone (2 mg/l), meropenem (2 mg/l) alone, combination of polymyxin-B/meropenem at their clinical breakpoints, and triple-antibiotic combination of polymyxin-B/meropenem and 4 mg/l sulbactam. The triple-antibiotic combination significantly changed the metabolite levels involved in cell outer membrane and cell wall biosynthesis, including fatty acid, glycerophospholipid, lipopolysaccharide, peptidoglycan, and nucleotide within 15 min of administration. In contrast, significant changes in metabolome were observed after 1 h in sample treated with either meropenem or polymyxin-B alone. After 1 h of administration, the double and triple combination therapies significantly disrupted nucleotide and amino acid biosynthesis pathways as well as the central carbon metabolism, including pentose phosphate and glycolysis/gluconeogenesis pathways, and tricarboxylic acid cycle. The addition of sulbactam to polymyxin-B and meropenem combination appeared to be an early disruptor of *A. baumannii* metabolome, which paves the way for further antibiotic penetration into bacteria cells. Combination antibiotics consisting of sulbactam/meropenem/polymyxin-B can effectively confer susceptibility to *A. baumannii* harboring OXA-23 and other drug resistant genes. Metabolomic profiling reveals underlying mechanisms of synergistic effects of polymyxin-B combined with meropenem and sulbactam against multi-drug resistant *A. baumannii.*

## Introduction

Multi-drug resistant (MDR) pathogens in nosocomial and community-acquired infections are an impending threat to public health. *Acinetobacter baumannii* infections is one of the most prevalent and fatal MDR pathogens often found in wound infection, urinary tract infections, empyema, pneumonia and meningitis (Tacconelli et al., 2018). Polymyxin antibiotics are increasingly being used as last resort against MDR *A. baumannii infections* since 2000s (Li et al., 2006; Poirel et al., 2017). The mechanism of action for polymyxins has been well studied. It was proposed that polymyxins disrupted the outer cell membrane by binding to the negatively charged lipopolysaccharide (LPS), the predominant surface lipid of the outer membrane in Gram-negative bacteria (Jiang et al., 2020; Zhao et al., 2021). However, *A. baumannii* can become highly resistant to polymyxins by incorporating phosphoethanolamine or galactosamine to lipid A structure or through the loss of LPS production (Moffatt et al., 2010; Pelletier et al., 2013; Maifiah et al., 2016; Han et al., 2018; Zhao et al., 2021).

Combination therapies using polymyxins and other antibiotics are recommended to treat MDR pathogens. Among β-lactamase inhibitors, sulbactam has the highest intrinsic bactericidal activity against *A. baumannii* and is usually combined with other antibiotics to treat MDR *A. baumannii* infections (Ripa et al., 1990; Gales et al., 1996; Pei et al., 2012). Previous studies have shown that polymyxin-B combined with meropenem and sulbactam could achieve favorable antibacterial effects against *A. baumannii* (Lenhard et al., 2017; Kulengowski et al., 2019; Menegucci et al., 2019; Fedrigo et al., 2021), but the mechanism for their synergism is yet to be explored. In this paper, we employed metabolomic profiling to elucidate the synergistic mechanism of polymyxin-B when combined with meropenem and sulbactam in the treatment of MDR *A. baumannii*.

## Materials and methods

### Antibiotics, reagents and bacterial isolates

Polymyxin-B sulfate, meropenem and sulbactam (Shanghai Macklin Biochemical Co. Ltd. Shanghai, China) solutions were prepared according to the guidance from The Clinical and Laboratory Standards Institute (CLSI). Briefly, the stock solutions of polymyxin-B, meropenem and sulbactam (concentration: 5120 mg/l for all three antibiotics) were dissolved in dimethyl sulfoxide (DMSO). The final DMSO concentration in the culture medium was less than 1/1000 (v/v). Working solutions were prepared in Milli-Q water (Millipore, North Rye, Australia) and filtered before use. Three *A. baumannii* clinical strains were isolated from patients at the Affiliated Hospital of Qingdao University in 2020 and grown in cation-adjusted Mueller-Hinton broth (CAMHB; Land Bridge, Beijing, China). *E. coli* ATCC 25922 and *A. baumannii* ATCC 19606 were used as a quality control strain for antimicrobial susceptibility tests. The isolate carries several known resistant genes (Table 1).

**Table 1:** Drug resistance genes of *Acinetobacter baumannii* isolates.

**Affected antibiotics**	**Genes**		
	Isolate F	Isolate 13	Isolate 20
Streptogramin b	*msr(E)*	*msr(E)*	*msr(E)*
Tetracycline	*tet(B)*	*tet(B)*	*tet(B)*
Folate pathway antagonist	*sul2*	*sul1*	*sul1*
Aminoglycoside	*armA; aph(3″)-Ib; aph(6)-ld; aph(3)-la;*	*armA; aac(6)-lb3; aac(6″)-lb-cr; aph(3″)-lb; aph(6)-ld*	*armA; aadA1; aph(6)-ld; aph(3′)-la; aph(3″)-b; aac(6′)-b-cr; aac(6″)-lb3*
Macrolide	*msr(E); mph(E)*	*msr(E); mph(E)*	*msr(E); mph(E)*
Beta-lactam	*bla*OXA-23; *bla*ADC-25; *bla*TEM-1D; *bla*OXA-66	*bla*OXA-23; *bla*ADC-25; *bla*TEM-1D; *bla*OXA-66	*bla*OXA-23; *bla*ADC-25; *bla*OXA-66

### Susceptibility testing

A checkerboard assay with the typical broth microdilution was used to determine the susceptibility profile of each clinical isolate to the three drugs either alone or in combination, according to CLSI guidelines (CLSI, 2020). Susceptibility determination was conducted in triplicate for each of the *A. baumannii* isolates in a sterile 96-well microdilution plate. A standard inoculum size of 0.5 McFarland was prepared and read using a nephelometer (bioMérieux, Marcy l’Etoile, France); the resulting inoculum was diluted into each well to achieve a final concentration of 5 × 10^5^ cfu/ml. The prepared plate was incubated at 35 ± 2°C for 20 h. The concentration ranges of polymyxin-B and meropenem alone and in combination were 1 to 64 mg/l and 1 to 128 mg/l, respectively. The concentration of sulbactam was fixed at 4 mg/l for the combination test, since ampicillin/sulbactam susceptible breakpoint is ≤8/4 mg/l (CLSI, 2020). Note that the triple-antibiotic combination in this context refers to sulbactam/meropenem/polymyxin-B.

Based on the results of the checkerboard assay, the fractional inhibitory concentration index (FICI) was calculated according to the following equation to classify the antimicrobial synergy of the combination, $\begin{array}{l} \mathrm{FICI}=\frac{MIC\;ofantibiotic1\;incombination}{MIC\;ofantibiotic1\;alone}+\\ \frac{MIC\;ofantibiotic2\;incombination}{MIC\;ofantibiotic2\;alone}+\\ \frac{MIC\;ofantibiotic3\;incombination}{MIC\;ofantibiotic3\;alone} \end{array}$ .

When FICI is ≤0.5, the two drugs are considered synergistic; FICI >0.5–1 is additive; >1–<2 indicates indifference; and ≥2 is antagonistic (Hall et al., 1983).

### Bacterial culture

Based on the susceptibility testing result, an *A. baumannii* isolate was selected for metabolomic profiling. This isolate was cultured on a nutrient agar plate from the frozen stock (−80°C) and incubated at 37°C. For the overnight culture, a single colony of *A. baumannii* was inoculated into 15 ml CAMHB and the content was incubated in a shaking water bath at 180 rpm and 37°C. To get sufficient cell number, the bacterial culture was grown to an optical density (OD_600_) of approximate 0.5 to achieve a starting inoculum at around 10^8^ CFU (colony forming units)/mL during an early exponential growth phase (Maifiah et al., 2017). The concentrations of polymyxin-B, meropenem and sulbactam were chosen based on their clinical breakpoints. Bacterial culture was treated with polymyxin-B (2 mg/l) and meropenem (2 mg/l) as both monotherapy and the combination of the two drugs (P + M, 2 mg/l + 2 mg/l, respectively), and the combination with sulbactam (P + M + S, 2 mg/l + 2 mg/l + 4 mg/l, respectively). Bacteria cultured without any antibiotic served as control. Five biological replicates were prepared for each treatment.

### Preparation of cellular metabolite extracts

Cellular metabolites of *A. baumannii* were extracted using previously reported method (Maifiah et al., 2016). Briefly, samples were centrifuged at 3,220 × g at 4°C for 20 min; the supernatant was discarded; and the bacterial pellets were washed twice with 1 ml of cold saline. Next, 500 μl of cold chloroform-methanol–water (CMW; 1,3:1, v/v/v) solution containing the internal standards, 3-[(3-cholamidopropyl)-dimethylammonio]-1-propanesulfonate (CHAPS), N-cyclo-hexyl-3-aminopropanesulfonic acid (CAPS), piperazine-N,N′-bis(2-ethanesulfonic acid; PIPES), and Tris at 1 μM, was added. The samples were flash frozen in liquid nitrogen, thawed on ice, and vortexed to release the intracellular metabolites. The samples were centrifuged for 10 min at 3,220 × g at 4°C to remove cell debris, and then 300 μl of the supernatants was added to 1.5 ml Eppendorf tubes. After centrifugation at 14,000 × g at 4°C for 10 min, 200 μl of supernatant was transferred into injection vials for bioanalysis. Quality control (QC) samples were obtained by pooling the samples and extracted as described above.

### LC–MS analysis

The LC–MS method was based on a previously reported method with modifications (Maifiah et al., 2017; Ribera et al., 2019; Zhao et al., 2021). Samples were analyzed by liquid chromatography-high resolution mass spectrometry (LC–MS) equipped with an Ultimate 3,000 ultra-high-performance liquid chromatography (UHPLC) system (Thermo Scientific, CA, United States) and Q-Exactive Orbitrap mass spectrometer (Thermo Scientific, CA, USA) operated in both positive and negative electrospray ionization (ESI) modes (polarity switching) at a resolution of 35,000 with a detection range of m/z 50 to 1,000 Da. The separation was performed on a HILIC column (2.1 × 100 mm, 1.7 μm, ACE 1.7 HILIC-A, United Kingdom) coupled with a guard column (ACE UHPLC Pre-column filter) operated at 40°C. The mobile phase which consisted of 10 mM ammonium carbonate in water (solvent A) and acetonitrile (solvent B) was delivered at a flow rate of 0.3 ml/min. The elution mode with an overall duration of 29 min started from 80% B transitioning to 20% B at 15 min, followed by a wash with 5% B for 3 min at 18 min, and a final re-equilibration for 8 min with 80% B. The injection volume was 10 μl.

### Data processing, bioinformatics and statistical analyses

**Figure 1:**
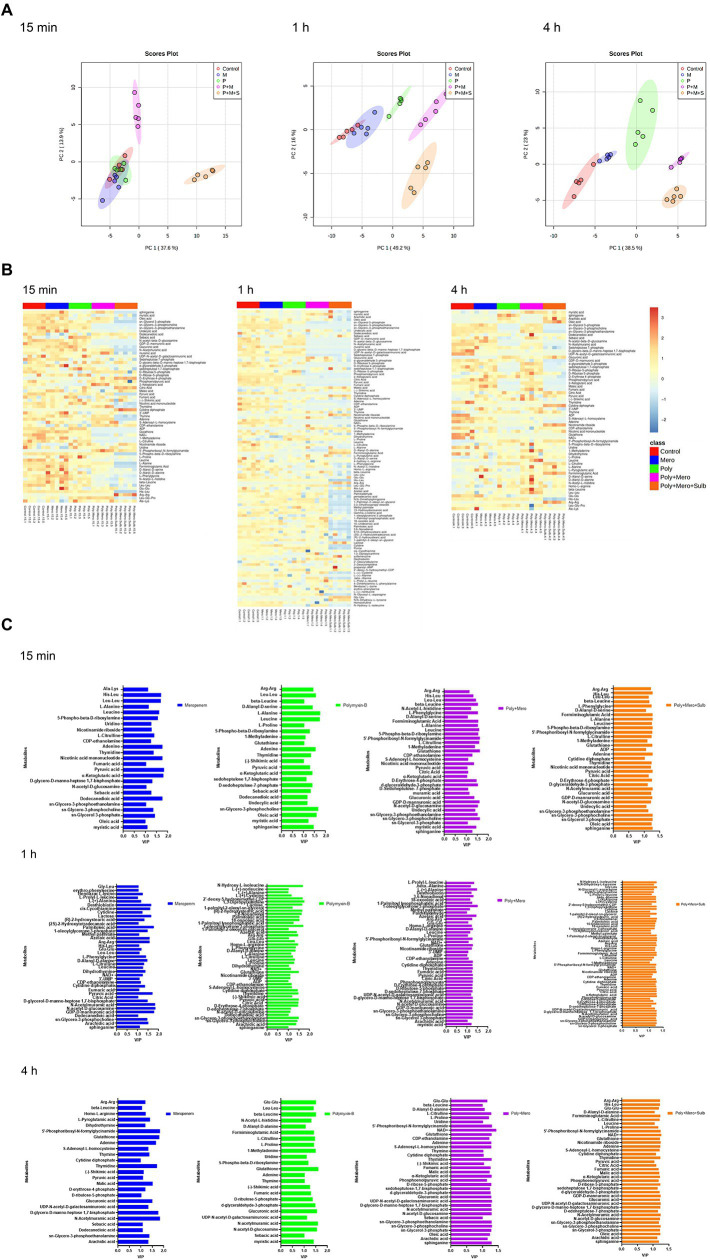
Perturbations of lipid metabolism pathways against *Acinetobacter baumannii* isolate treated with antibiotics as monotherapy and combination therapy at 15 min, 1 h and 4 h. Significantly perturbed metabolites were selected based on log_2_FC ≤ −1 or  ≥ 1, *p* < 0.05 and VIP > 1; *p < 0.05; **p < 0.01; ***p < 0.001.

The raw data file obtained from the LC–MS was processed and analyzed by Progenesis QI program (Waters). Briefly, the workflow was established for importing data, automatic alignment, peak selection based on minimum intensity of 50,000, and subsequently identifying these metabolites. Metabolites were characterized by LC retention time and accurate *m/z* value. The maximum retention time shift for peak alignment was limited to 0.2 min and the mass tolerance was 5 ppm. Metabolite intensities were normalized by the sum, followed by log_10_-transformation and auto scaling (mean-centered divided by standard deviation) of individual values. Statistical analysis was performed using MetaboAnalyst 5.0 web portal.^1^ Principal component analysis (PCA) was performed for all treatment groups at each time point (Figure 1). Student’s t-test was used to determine metabolites with significantly changed intensity [*p* < 0.05, fold change (FC) ≥2 (log_2_FC ≥ 1 or ≤ −1) and variable important in projection (VIP > 1)]. Pathway analysis was performed using KEGG (Kyoto Encyclopedia of Genes and Genomes) and HMDB (The Human Metabolome Database) databases (Maifiah et al., 2017; Ribera et al., 2019; Zhao et al., 2021).

## Results

### *In vitro* antimicrobial susceptibility

All three *A. baumannii* isolates carried OXA-23 along with other drug-resistance genes (Table 1) and showed significant drug resistance to sulbactam, polymyxin-B and meropenem (Table 2). The MIC of meropenem alone against these isolates ranged from 32 to >128 mg/l, whereas the MIC of polymyxin-B alone ranged from 8 to 16 mg/l. MIC of sulbactam alone was ≥64 mg/l. The Clinical Laboratory Standards Institute (CLSI) breakpoints were used for the interpretation of polymyxin-B MIC results: ≤2 mg/l (intermediate), >2 mg/l (resistant); and meropenem MIC results: ≤2 mg/l (susceptible), 4 mg/l (intermediate), and ≥ 8 mg/l (resistant) for *A. baumannii* (CLSI, 2020). These isolates are considered resistant to both meropenem and polymyxin-B. Even though there is no interpretive breakpoint for sulbactam, these isolates can be considered resistant to sulbactam, since the clinical regimens could not sufficiently achieve drug concentration of 64 mg/l in the blood.

**Table 2:** Minimum inhibitory concentrations of meropenem, polymyxin-B and sulbactam alone or in combination with or without sulbactam (4 mg/L) against carbapenem-resistant *Acinetobacter baumannii* isolates and fractional inhibitory concentration index (sulbactam was fixed at 4 mg/L).

Strains	**MIC (mg/L)**							**Synergism analysis**	
	Monotherapy			Combination therapy					
	Meropenem	Polymyxin-B	Sulbactam	Meropenem/sulbactam	Polymyxin-B/ sulbactam	Meropenem/ polymyxin-B	Meropenem/polymyxin-B/sulbactam	FIC index^‡^	FICI category
*E. coli* ATCC25922	1	1	32						
*A. baumannii*									
F	>128	8	>64	>128/4	8/4	8/2	2/2/4	0.3281	Synergism
13	64	16	>64	64/4	16/4	8/2	2/2/4	0.2188	Synergism
20	32	8	>64	32/4	8/4	2/2	≤1/2/4	0.3438	Synergism

MIC, minimum inhibitory concentration; FIC, fractional inhibitory concentration.

‡ FIC index was computed using the reduced MICs of meropenem, polymyxin-B and sulbactam in the triple-antibiotic combination relative to meropenem, polymyxin-B and sulbactam monotherapies. CLSI breakpoints for interpretation of polymyxin-B MIC results: ≤2 mg/l (intermediate), >2 mg/l (resistant); and meropenem MIC results: ≤2 mg/l (susceptible), 4 mg/l (intermediate), and ≥ 8 mg/l (resistant) for *A. baumannii*.

The combination of polymyxin-B and meropenem reduced the MIC of polymyxin-B and meropenem to lower than their breakpoints (2 mg/l) in 1/3 strains, and the FICI scores were less than 0.5 for all strains. The addition of 4 mg/l sulbactam to the meropenem/polymyxin-B combination further lowered meropenem MIC values to ≤2 mg/l in all isolates, and the FICI scores of isolate 13 was 0.2188 which was the lowest FICI score of the 3 isolates. Based on the results from *in vitro* antimicrobial susceptibility, isolate 13 was selected for the metabolomic study.

### Metabolomic changes in *Acinetobacter baumannii* after treatment with polymyxin-B, meropenem as monotherapy, and in combination with sulbactam

**Figure 2:**
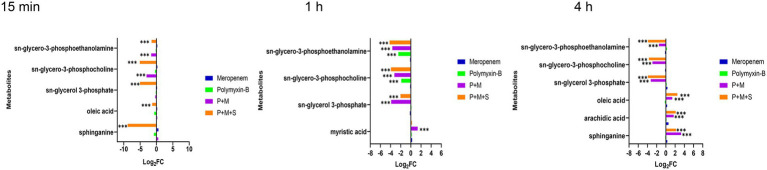
Principal component analysis **(A)**, Heatmap **(B)**, and variable important in projection >1 **(C)** profile of MDR *Acinetobacter baumannii* treated with meropenem and polymyxin-B as monotherapy and in combination with and without sulbactam at 15 min, 1 h and 4 h.

Metabolomic analysis using LC–MS identified a total number of 222 metabolites. The median relative standard deviations (RSD) for metabolites detected in QC samples were less than 10.0%, indicating that the analysis is highly reproducible. A PCA was performed to delineate putative metabolites that contributed to differential effects of drug treatments on *A. baumannii*. In samples treated with drugs for 15 min, clear differences were observed between combination therapy (P + M and P + M + S), monotherapy and control group; significant metabolite alterations were observed in response to P + M and P + M + S (Figure 2A). In contrast, the metabolome of polymyxin-B and meropenem monotherapy groups overlapped with the control group. The heatmap and VIP showed overall changes in metabolite levels and important differential metabolites (VIP > 1), respectively (Figures 2B,C).

A total of 12 significant metabolites whose abundance had changed in the P + M groups at 15 min were identified: 11 metabolites decreased in abundance after exposure to combination therapy, while 1 metabolite exhibited increased levels. For P + M + S group, 28 metabolites were altered at 15 min, of which 24 decreased and 4 increased. After 1 h of treatment, polymyxin-B and meropenem alone induced minor metabolomic changes, while combined drug treatments led to significant alterations (Figure 1); a total number of 25 (2 increased, 23 decreased compared with the control group) and 49 metabolites (6 increased, 43 decreased compared with the control group) were changed in response to P + M and P + M + S treatments, respectively. At 4 h, a total number of 27 (7 increased, 20 decreased compared with the control group) and 30 metabolites (9 increased, 21 decreased compared with the control group) were significantly perturbed by P + M and P + M + S treatments, respectively. All altered metabolites were grouped accordingly to correlate with metabolic pathways, which were assigned to lipids, lipopolysaccharide, amino sugar, peptidoglycan, nucleotide, amino acids, and carbohydrate metabolisms.

### Polymyxin-B plus meropenem with or without sulbactam treatment disrupted lipid metabolic pathway in *Acinetobacter baumannii*

Lipid metabolic pathways including glycerophospholipid, fatty acids, and sphingolipid were affected by the combination therapies. The levels of three metabolites including sn-glycerol-3-phosphate, sn-glycero-3-phosphocholine and sn-glycero-3-phosphoethanolamine (log_2_FC = −5.1 to-1.5), which are important intermediates for membrane structure, were significantly downregulated after the combination treatments at the three time points. In addition, the P + M + S combination remarkably perturbed an intermediate related to sphingolipid pathway which is sphinganine (log_2_FC = −8.8 and 2.2) at 15 min and 4 h.

Fatty acid pathway was also significantly affected by the P + M + S treatment. The level of oleic acid was decreased at 15 min but increased after 4 h of treatment, and arachidic acid was remarkably increased at 4 h. Polymyxin-B and meropenem monotherapies did not significantly induce these changes in lipid metabolism within the investigation period (Figure 1).

### Polymyxin-B plus meropenem with or without sulbactam treatment affect lipopolysaccharide, peptidoglycan and amino sugar metabolisms

**Figure 3:**
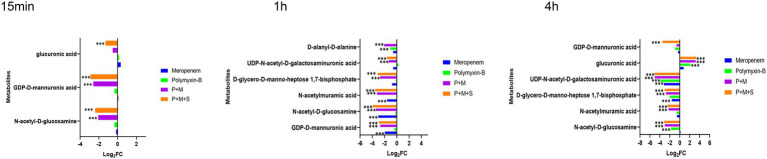
Perturbations of LPS and peptidoglycan metabolism pathway in *Acinetobacter baumannii* treated with antibiotics as monotherapy and combination therapy at 15 min, 1 and 4 h. Significantly perturbed metabolites were selected based on log_2_FC ≤ −1 or  ≥ 1, *p* < 0.05 and VIP > 1; *p < 0.05; **p < 0.01; ***p < 0.001.

Lipopolysaccharide (LPS), peptidoglycan and amino sugar are essential components of the outer membrane of gram-negative bacteria. The levels of intermediate metabolites associated with LPS, peptidoglycan and amino sugar biosynthesis pathways were changed after treatment with combination therapy (Figure 3). The P + M and P + M + S combinations significantly induced changes in the level of N-acetyl-D-glucosamine (GLcNAc) and GDP-D-mannuronic acid (GDP-ManA), which are two important intermediates associated with amino sugar biosynthesis pathway (log_2_FC = −3.9 to 2.2) at the three time points. In addition, N-acetylmuramic acid (MurMAc), another metabolite related to amino sugar pathway, was perturbed by the combination therapy at 1 h and 4 h (log_2_FC = −2.5 to-3.5). Significant downregulations of D-glycero-D-manno-heptose-1,7-bisphosphate and UDP-N-acetyl-D-galactosaminuronic acid (UDP-GalNAcA; log_2_FC = −5.0 to-1.6) were observed at 1 h and 4 h, which are related to LPS and peptidoglycan pathways, respectively. The level of glucuronic acid was perturbed by P + M + S at 15 min and 4 h. The changes induced by P + M + S treatment were similar to that of P + M group, indicating that sulbactam treatment did not exacerbate metabolite alterations induced by P + M in *A. baumannii*. Compared to the combination therapy, monotherapies of polymyxin-B and meropenem did not perturb the level of metabolites at 15 min. Meropenem alone significantly decreased the levels of GDP-D-mannuronic acid, N-acetyl-D-glucosamine and N-acetylmuramic acid (log_2_FC = −3.0 to-1.6) at 1 h, and D-glycero-manno-heptose-1,7-bisphosphate and UDP-N-acetyl-D-galactosaminuronic acid (log_2_FC = −1.6/−3.2) at 4 h. Polymyxin-B alone only changed the levels of N-acetyl-D-glucosamine, UDP-N-acetyl-D-galactosaminuronic acid and glucuronic acid (log_2_FC = −3.9 to 2.1) at 4 h. The results showed that combination treatments resulted in a larger metabolomic change compared to monotherapy.

### Polymyxin-B plus meropenem with or without sulbactam treatment perturbed central carbon metabolism pathway

**Figure 4:**
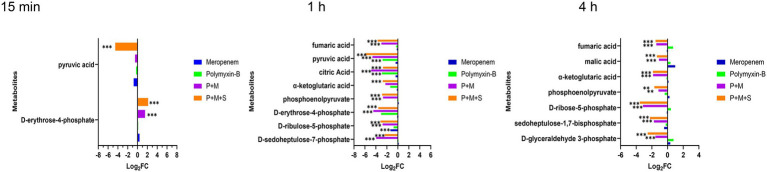
Perturbations of central carbon metabolism pathway in *Acinetobacter baumannii* treated with antibiotics as monotherapy and combination therapy at 15 min and 1 h. Significantly perturbed metabolites were selected based on log_2_FC ≤ −1 or  ≥ 1, p < 0.05 and VIP > 1; *p < 0.05; **p < 0.01; ***p < 0.001.

After a 15 min treatment, the two-drug combination induced an increase in D-erythrose-4-phosphate (log_2_FC = 1.5/1.2) while P + M + S combination also decreased the level of pyruvic acid (log_2_FC = 1.3). Meropenem or polymyxin alone did not induce significant changes in the central carbon metabolism pathway. At 1 h and 4 h, the metabolite types and level disorders caused by the combination of P + M and P + M + S were similar (Figure 4). Five metabolites involved in tricarboxylic acid cycle (TCA) pathway including phosphoenolpyruvate, α-ketoglutaric acid, citric acid, fumaric acid and pyruvic acid (log_2_FC = −5.9 to-2.7) were significantly downregulated by the two combination treatment groups at 1 h. At the same time, the levels of D-sedoheptulose-7-phosphate, D-ribulose-5-phosphate and D-erythrose-4-phosphate (log_2_FC = −4.5 to-2.4), which are three intermediates associated with pentose phosphate pathway (PPP) were also decreased by P + M and P + M + S treatments. In addition, the disruption of another three metabolites associated with PPP including D-glyceraldehyde-3-phosphate, sedoheptulose-1,7-bisphosphate and D-ribose-5-phosphate (log_2_FC = −3.5 to-1.5) were also observed at 4 h after either P + M or P + M + S treatments. Among the above metabolites, D-sedoheptulose-7-phosphate and D-ribulose-5-phosphate were also involved in the LPS pathway. After 4 h treatment of either P + M or P + M + S, the altered metabolites involved in TCA cycle were mainly phosphoenolpyruvate, α-ketoglutaric acid, malic acid and fumaric acid (log_2_FC = −1.9 to-1.1). For monotherapy, polymyxin-B alone affected the levels of citric acid and pyruvic acid (log_2_FC = −2.9/−2.8) at 1 h whereas treatment with meropenem alone was not associated with significant changes in metabolomic profiles.

### Polymyxin-B plus meropenem with or without sulbactam treatments altered nucleotide, nicotinate and nicotinamide, amino acid and peptide metabolism pathways

**Figure 5:**
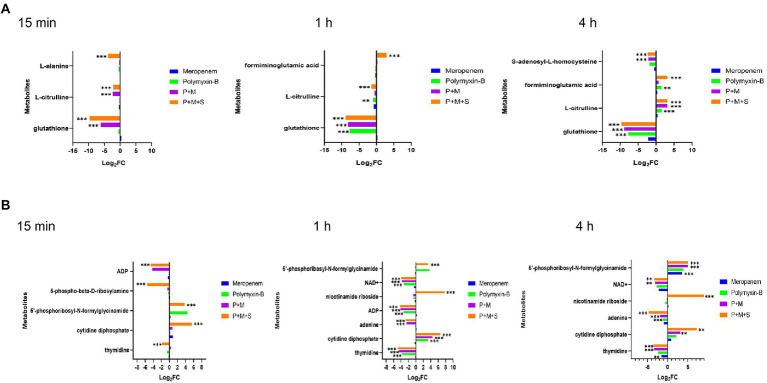
Perturbations of amino acid **(A)** and nucleotide **(B)** metabolism pathways in *Acinetobacter baumannii* treated with antibiotics as monotherapy and combination therapy at 15 min, 1 h and 4 h. Significantly perturbed metabolites were selected based on log_2_FC ≤ −1 or  ≥ 1, *p* < 0.05 and VIP > 1; *p < 0.05; **p < 0.01; ***p < 0.001.

The combination therapy altered both the pyrimidine and purine metabolisms within 15 min of treatment (Figure 5). At the three time points, P + M + S treatment disturbed the equilibrium of thymidine (log_2_FC = −4.9 to-1.8) and cytidine diphosphate (CDP, log_2_FC = 5.6 to 7.2), two important metabolites associated with pyrimidine metabolism pathway, as well as levels of adenine (log_2_FC = −5.7 to-2.7) and 5′-phosphoribosyl-N-formylglycinamide (FGAR, log_2_FC = 3.3 to 5.1) which are related to purine metabolism. ADP (log_2_FC = −4.6 to-4.3) was significantly downregulated by the triple combination treatment at 15 min and 1 h.

The metabolite disequilibrium caused by P + M was similar in both the affected metabolites and their intensities to that due to P + M + S. The combination of P + M and P + M + S significantly reduced the level of NAD^+^ (log_2_FC = −3.9 to-3.1) at 1 h and 4 h. The P + M + S group induced a change in glutathione (GSH) and L-citrulline (log_2_FC = −9.7 to 3.1) at the three time points. For polymyxin-B monotherapy treatment, the levels of thymidine, CDP, ADP, glutathione, NAD^+^ and L-citrulline (log_2_FC = −7.7 to 3.3) were altered at 1 h, while at 4 h, the levels of adenine, glutathione, L-citrulline and formiminoglutamic acid (log_2_FC = −7.7 to 1.6) were affected. Meropenem alone only affected the levels of thymidine, glutathione and 5′-phosphoribosyl-N-formylglycinamide (log_2_FC = −2.1 to 3.6) at 4 h.

## Discussion

The World Health Organization lists MDR Gram-negative bacteria as a critical threat to vulnerable patients (Van Duin et al., 2013; Zilberberg et al., 2017). Polymyxins are regarded as a treatment of last resort against life-threatening MDR Gram-negative bacteria, but recent usage pattern of polymyxins has led to increasing resistance of pathogens to polymyxins (Srinivas and Rivard, 2017).

The present study applied metabolomic profiling to investigate the mechanisms underlying the synergistic effects of meropenem/polymyxin-B/sulbactam against MDR *A. baumannii.* These isolates harbor OXA-23, TEM and ADC β-lactamases, which confer resistance to both meropenem and sulbactam. OXA-23, in particular, is not inhibited by sulbactam and is a prevalent mechanism contributing to sulbactam resistance in diverse *A. baumannii* clinical isolates (Yang et al., 2019). The combination of meropenem and polymyxin-B could significantly reduce the MIC values but only to 8/2 mg/l in two isolates, which are considered resistant to meropenem and intermediate to polymyxin-B. In the third isolate, meropenem/polymyxin-B MICs were 2/2 mg/l. 4 mg/l sulbactam further reduces MIC to ≤2/2/4 mg/l for meropenem/polymyxin-B/sulbactam, resulting in susceptible for meropenem and intermediate for polymyxin-B (CLSI, 2020). The reduction in MIC provides sufficient drug exposure for clinical regimens of meropenem, polymyxin-B and sulbactam to achieve ≥90% PTA for their respective pharmacodynamic indices (Martins et al., 2020; Zhu et al., 2022). The metabolomic profiling results revealed that the combination therapy (P + M + S) initially perturbed lipids, LPS and peptidoglycan metabolism therefore impacted the stability of cell membrane and cell wall. This effect was followed by the disturbance of the central carbon metabolism (CCM) and nucleotide metabolism in the cell, which co-occurred with oxidative stress as demonstrated by the depletion of intracellular antioxidant GSH. The sequence of events is summarized in Table 3.

**Table 3:** Sequence of metabolomic changes in *Acinetobacter baumannii* after polymyxin-B and meropenem treatments as monotherapy and in combination with and without sulbactam.

**Time**	**Polymyxin-B/Meropenem/Sulbactam**	**Polymyxin-B/Meropenem**	**Polymyxin-B**	**Meropenem**
**15 min**	Cell wall synthesis ↓ N-acetyl-D-glucosamine; ↓ GDP-D-mannuronic acid	Cell wall synthesis ↓ N-acetyl-D-glucosamine; ↓ GDP-D-mannuronic acid	Cell wall synthesis NA	Cell wall synthesis NA
	Outer membrane glycerophospholipids ↓ sn-glycerol-3-phosphate; ↓ sn-glycero-3-phosphocholine; ↓ sn-glycero-3-phosphoethanolamine	Outer membrane glycerophospholipids ↓ sn-glycero-3-phosphocholine; ↓ sn-glycero-3-phosphoethanolamine	Outer membrane glycerophospholipids NA	Outer membrane glycerophospholipids NA
	Central carbon metabolism pathway ↑ D-erythrose-4-phosphate; ↓ pyruvic acid	Central carbon metabolism pathway ↑ D-erythrose-4-phosphate	Central carbon metabolism pathway NA	Central carbon metabolism pathway NA
	Nucleotide, nicotinate and nicotinamide, amino acid and peptide pathway ↓ L-alanine; ↓ L-citrulline; ↓ glutathione; ↓ 5-phospho-beta-D-ribosylamine; ↑ 5′-phosphoribosyl-N-formylglycinamide; ↑ cytidine diphosphate; ↓ thymidine;↓ADP	Nucleotide, nicotinate and nicotinamide, amino acid and peptide pathway ↓ L-alanine; ↓ L-citrulline; ↓ glutathione; ↓ ADP	Nucleotide, nicotinate and nicotinamide, amino acid and peptide pathway NA	Nucleotide, nicotinate and nicotinamide, amino acid and peptide pathway NA
**1 h**	Cell wall synthesis ↓ N-acetyl-D-glucosamine; ↓ N-acetylmuramic acid; ↓ GDP-D-mannuronic acid; ↓ UDP-N-acetyl-D-galactosaminuronic acid; ↓ D-glycero-beta-D-manno-heptose 1,7-bisphosphate	Cell wall synthesis ↓ N-acetyl-D-glucosamine; ↓ N-acetylmuramic acid; ↓ GDP-D-mannuronic acid; ↓ UDP-N-acetyl-D-galactosaminuronic acid; ↓ D-glycero-beta-D-manno-heptose 1,7-bisphosphate; ↓ D-alanyl-D-alanine	Cell wall synthesis NA	Cell wall synthesis ↓ N-acetyl-D-glucosamine; ↓ GDP-D-mannuronic acid; ↓ N-acetylmuramic acid
	Outer membrane glycerophospholipids ↓ sn-glycerol-3-phosphate; ↓ sn-glycero-3-phosphocholine; ↓ sn-glycero-3-phosphoethanolamine	Outer membrane glycerophospholipids ↑ myristic acid; ↓ sn-glycerol-3-phosphate; ↓ sn-glycero-3-phosphocholine; ↓ sn-glycero-3-phosphoethanolamine	Outer membrane glycerophospholipids ↓ sn-glycerol-3-phosphate; ↓ sn-glycero-3-phosphocholine; ↓ sn-glycero-3-phosphoethanolamine	Outer membrane glycerophospholipids ↓ sn-glycerol-3-phosphate; ↓ sn-glycero-3-phosphocholine; ↓ sn-glycero-3-phosphoethanolamine
	Central carbon metabolism pathway ↓ fumaric acid; ↓ pyruvic acid; ↓ citric acid; ↓ α-ketoglutaric acid; ↓ phosphoenolpyruvate; ↓ D-erythrose-4-phosphate; ↓ D-ribulose-5-phosphate; ↓ D-sedoheptulose-7-phosphate	Central carbon metabolism pathway ↓ fumaric acid; ↓ pyruvic acid; ↓ citric acid; ↓ phosphoenolpyruvate; ↓ D-erythrose-4-phosphate; ↓ D-ribulose-5-phosphate; ↓ D-sedoheptulose-7-phosphate	Central carbon metabolism pathway ↓ pyruvic acid; ↓ citric acid	Central carbon metabolism pathway ↓ D-ribulose-5-phosphate
	Nucleotide, nicotinate and nicotinamide, amino acid and peptide pathway ↑ formiminoglutamic acid; ↓ L-citrulline; ↓ glutathione; ↓ NAD+; ↓ thymidine; ↑ 5′-phosphoribosyl-N-formylglycinamide; ↑ nicotinamide riboside; ↓ ADP; ↓ adenine; ↑ cytidine diphosphate	Nucleotide, nicotinate and nicotinamide, amino acid and peptide pathway ↓ glutathione; ↓ NAD+; ↓ ADP; ↓ adenine; ↓ thymidine; ↑ cytidine diphosphate	Nucleotide, nicotinate and nicotinamide, amino acid and peptide pathway ↓ L-citrulline; ↓ glutathione; ↓ NAD+; ↓ ADP; ↓ thymidine; ↑ cytidine diphosphate	Nucleotide, nicotinate and nicotinamide, amino acid and peptide pathway NA
**4 h**	Cell wall synthesis ↓ GDP-D-mannuronic acid; ↓ UDP-N-acetyl-D-galactosaminuronic acid; ↓ D-glycero-beta-D-manno-heptose 1,7-bisphosphate; ↓ N-acetylmuramic acid; ↓ N-acetyl-beta-D-glucosamine	Cell wall synthesis ↓ UDP-N-acetyl-D-galactosaminuronic acid; ↓ D-glycero-beta-D-manno-heptose 1,7-bisphosphate; ↓ N-acetylmuramic acid; ↓ N-acetyl-beta-D-glucosamine	Cell wall synthesis ↓ UDP-N-acetyl-D-galactosaminuronic acid; ↓ N-acetyl-beta-D-glucosamine	Cell wall synthesis ↓ UDP-N-acetyl-D-galactosaminuronic acid; ↓ D-glycero-beta-D-manno-heptose 1,7-bisphosphate
	Outer membrane glycerophospholipids ↓ sn-glycerol-3-phosphate; ↓ sn-glycero-3-phosphocholine; ↓ sn-glycero-3-phosphoethanolamine	Outer membrane glycerophospholipids ↓ sn-glycerol-3-phosphate; ↓ sn-glycero-3-phosphocholine; ↓ sn-glycero-3-phosphoethanolamine	Outer membrane glycerophospholipids NA	Outer membrane glycerophospholipids NA
	Central carbon metabolism pathway ↓ fumaric acid; ↓ maleic acid; ↓ α-ketoglutaric acid; ↓ phosphoenolpyruvate; ↓ D-ribose-5-phosphate; ↓ sedoheptulose-1,7-bisphosphate; ↓ D-glyceraldehyde-3-phosphate	Central carbon metabolism pathway ↓ fumaric acid; ↓ malic acid; ↓ α-ketoglutaric acid; ↓ phosphoenolpyruvate; ↓D-ribose-5-phosphate; ↓ sedoheptulose-1,7-bisphosphate; ↓ D-glyceraldehyde-3-phosphate	Central carbon metabolism pathway NA	Central carbon metabolism pathway NA
	Nucleotide, nicotinate and nicotinamide, amino acid and peptide pathway ↓ S-adenosyl-L-homocysteine; ↑ L-citrulline; ↑ formiminoglutamic acid; ↓ glutathione; ↓ NAD+; ↑ cytidine diphosphate; ↓ adenine; ↑ 5′-phosphoribosyl-N-formylglycinamide; ↑ nicotinamide riboside; ↓ thymidine	Nucleotide, nicotinate and nicotinamide, amino acid and peptide pathway ↓ S-adenosyl-L-homocysteine; ↑ L-citrulline; ↓ glutathione; ↓ NAD+; ↓ adenine; ↓thymidine; ↑ cytidine diphosphate; ↑ 5′-phosphoribosyl-N-formylglycinamide	Nucleotide, nicotinate and nicotinamide, amino acid and peptide pathway ↑ formiminoglutamic acid; ↑ L-citrulline; ↓ glutathione; ↓ adenine	Nucleotide, nicotinate and nicotinamide, amino acid and peptide pathway ↑ 5′-phosphoribosyl-N-formylglycinamide; ↓ thymidine

**Figure 6:**
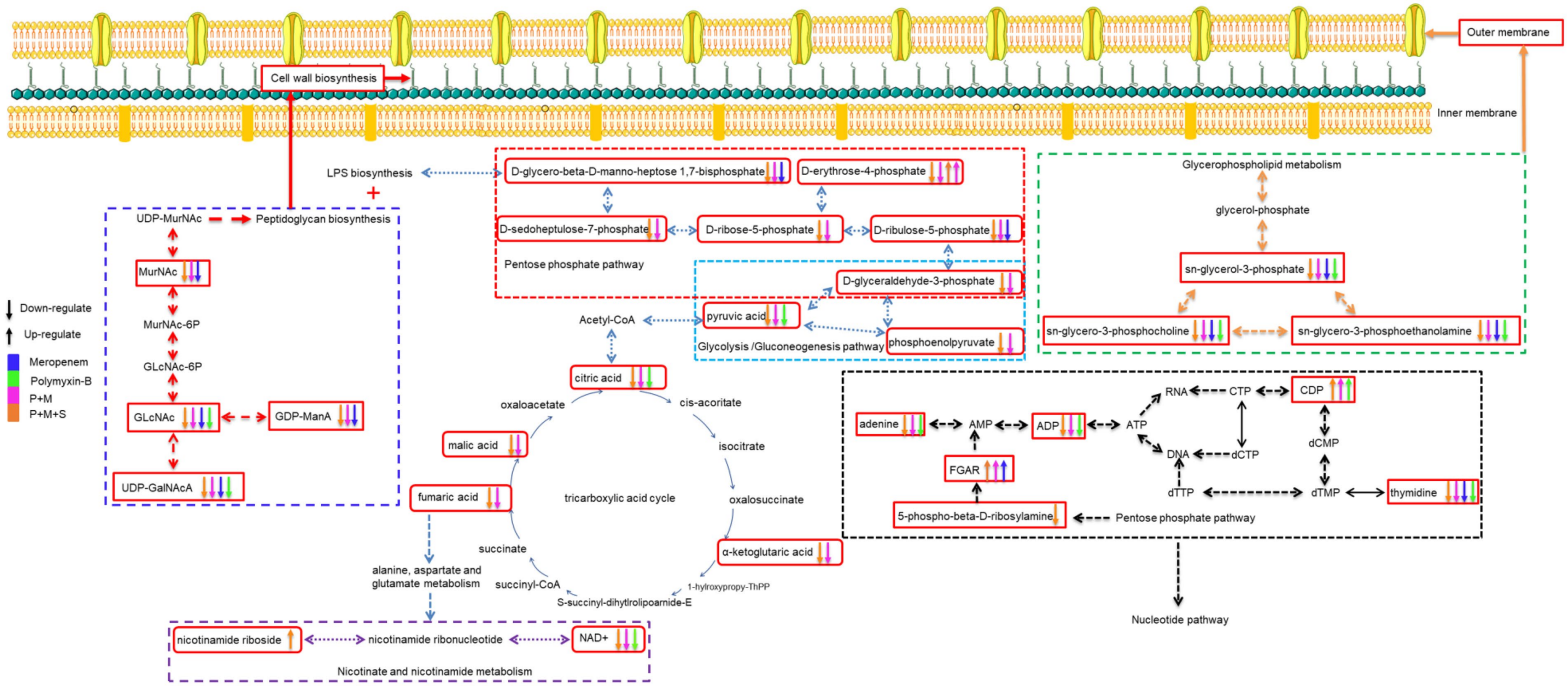
Illustration of affected metabolomic pathways in *Acinetobacter baumannii* treated with antibiotics as monotherapy and combination therapy.

An illustration of affected metabolic pathways is shown in Figure 6. The effect of sulbactam in disrupting metabolic pathways occurred at an earlier time as compared to P + M. Sulbactam effect on *A. baumannii* is accomplished by cell wall disintegration and cell lysis while also acting as a β-lactamase inhibitor (Horii et al., 2002; Lin et al., 2014; Penwell et al., 2015). Meropenem plays a role in cell wall thinning in the exposed bacteria through its high-affinity binding with penicillin-binding protein, which is a major cell wall synthesis enzyme (Cottagnoud, 2002; Sauvage et al., 2008; Bian et al., 2021). The cell wall of bacteria is mainly composed of lipopolysaccharide and peptidoglycan. The combination consisting of P + M and P + M + S depleted the levels of two important cell wall components that are GlcNAc and MurNAc. The levels of GDP-ManA and UDP-GalNAcA, which are two important intermediates involved in peptidoglycan biosynthesis, were in disequilibrium.

The levels of three metabolites related to pentose phosphate pathway were also affected by P + M and P + M + S: D-ribulose-5-phosphate, D-sedoheptulose-7-phosphate and D-glycero-beta-D-manno-heptose-1,7-bisphosphate; they are precursors of LPS biosynthesis (Maifiah et al., 2017; Hu et al., 2019). This shows that an early effect of sulbactam in disrupting and puncturing cell wall structure is an important contributor to the collapse of cell morphology (Horii et al., 2002). When the integrity and stability of cell membrane are disrupted, the therapeutic pressure can either eradicate the bacteria or prompt the bacteria to increase its defense by enhancing drug resistance mechanisms. Previous comparative metabolomic study evaluated sulbactam/colistin combination (Han et al., 2019a). This study showed that colistin/sulbactam combination decreased amino acid and nucleotide levels more dramatically at 4 h compared with monotherapies of either sulbactam or colistin; cell wall synthesis was also perturbed early on at 1 h by sulbactam.

Polymyxin-B effect on bacterial outer membrane is mediated by an electrostatic interaction with the lipid A of LPS and subsequently affected the stability of cell membrane (Cajal et al., 1996; Clausell et al., 2003; Domingues et al., 2012). Polymyxin-resistance in *A. baumannii* was previously shown to be mediated by LPS deficiency and lipid A modifications as well as a shift in glycerophospholipid profile that increased the abundance of short-chain lipid, resulting in a significant outer-membrane remodeling (Maifiah et al., 2016). The current study was not designed to compare polymyxin-resistant against polymyxin-susceptible *A. baumannii*. The combination of P + M and P + M + S reduced the levels of some metabolites associate with the synthesis of outer membrane glycerophospholipids including sn-glycerol-3-phosphate, sn-glycero-3-phosphocholine and sn-glycero-3-phosphoethanolamine (Zhang and Rock, 2008; Allobawi et al., 2020). Metabolites related to cell wall and outer membrane synthesis had only slight alteration when administered polymyxin-B and meropenem alone. This is likely due to the existing resistance mechanisms already at work in the clinical isolate.

The destruction of cell wall and outer membrane by the combination treatment changed the permeability of the outer membrane, allowing for more antibiotics to enter the bacteria (Nikaido, 2003; Khondker and Rheinstadter, 2020). Previous study showed interaction between transcriptomics and metabolomics in response to polymyxin treatment (Han et al., 2019b). In response to disruption of the outer membrane phospholipids by polymyxin, *P. aeruginosa* increased the biosynthesis of LPS and peptidoglycan to stabilize the damage to its cell envelope. The same feedback mechanism was also observed in *A. baumannii* (Maifiah et al., 2017). Subsequent CCM pathway is recruited to generate metabolic precursors in bacteria (Noor et al., 2010).

With more antibiotics entering the bacteria, the CCM pathway in the bacteria was also affected and many metabolites were in disequilibrium (Figure 4). The CCM is an important pathway that can provide energy; disruption of this pathway affects the bacteria’s oxidation and reduction states that can modify precursor metabolites for other metabolic pathways (Gest, 1987; Fuchs et al., 2012; Murima et al., 2014; Wolfe, 2015).

The stability of CCM directly affects the survival of bacteria cells (Lobritz et al., 2015). Twelve key metabolites in CCM were perturbed by the combination of P + M and P + M + S (Figure 4). Among them, fumaric acid is involved in the nicotinate and nicotinamide metabolism. The alterations in fumaric acid levels might lead to the perturbation of NAD+ and nicotinamide riboside. NAD+ and ADP which are markers of energy metabolism were depleted by P + M and P + M + S combination therapies. Depletion of NAD+ is a common strategy to enhance immune-mediated cell death in both prokaryotes and eukaryotes (Wang et al., 2022).

The nucleotide metabolism pathway is essential for energy, lipid and protein biosynthesis (Moffatt and Ashihara, 2002; Armenta-Medina et al., 2014; Zhao et al., 2021). This pathway was perturbed by the P + M + S combination treatment. Many metabolites were depleted including ADP, thymidine, adenine, 5-phospho-beta-D-ribosylamine, FGAR and CDP.

Glutathione as an integral part of cellular redox system is a key indicator of oxidative stress (Smirnova and Oktyabrsky, 2005). The perturbation of amino acid and peptide biosynthesis pathway was observed with the combination of P + M and P + M + S, especially on glutathione levels. The significantly depleted glutathione is consistent with the use of glutathione pool to compensate for antibiotic-induced oxidative damage (Maifiah et al., 2017). This shows that the metabolic balance in bacteria has been broken, thus inhibiting bacterial growth.

In conclusion, our findings were consistent with reported *in vitro* benefits of combination therapy in the treatment of MDR *A. baumannii* (Menegucci et al., 2019). The current study on metabolomic profiling elucidates the salutary effects of combination antimicrobial chemotherapy. The addition of sulbactam to P + M combination appears to be an early disruptor of *A. baumannii* metabolome, which paves the way for further antibiotic penetration into bacteria cells.

## Data availability statement

All authors listed have made a substantial, direct, and intellectual contribution to the work and approved it for publication.

## Author contributions

All authors listed have made a substantial, direct, and intellectual contribution to the work and approved it for publication.

## Funding

This study was supported by a grant from Shandong Provincial Natural Science Foundation (ZR2019BC025).

## Conflict of interest

The authors declare that the research was conducted in the absence of any commercial or financial relationships that could be construed as a potential conflict of interest.

## Publisher’s note

All claims expressed in this article are solely those of the authors and do not necessarily represent those of their affiliated organizations, or those of the publisher, the editors and the reviewers. Any product that may be evaluated in this article, or claim that may be made by its manufacturer, is not guaranteed or endorsed by the publisher.

## Footnotes

^1^ https://www.metaboanalyst.ca/
